# A Re-Purposing Strategy: Sub-Lethal Concentrations of an Eicosanoid Derived from the Omega-3-Polyunsaturated Fatty Acid Resolvin D1 Affect Dual Species Biofilms

**DOI:** 10.3390/ijms241612876

**Published:** 2023-08-17

**Authors:** Angela Maione, Annalisa Buonanno, Marilena Galdiero, Elisabetta de Alteriis, Francesco Petrillo, Michele Reibaldi, Marco Guida, Emilia Galdiero

**Affiliations:** 1 Department of Biology, University of Naples ‘Federico II’, Via Cinthia, 80126 Naples, Italy; angela.maione@unina.it (A.M.); annalisa.buonanno@unina.it (A.B.); dealteri@unina.it (E.d.A.); marco.guida@unina.it (M.G.); 2 Department of Experimental Medicine, University of Campania “Luigi Vanvitelli”, 81100 Naples, Italy; marilena.galdiero@unicampania.it; 3 Department of Medical Sciences, Eye Clinic, Turin University, 10126 Turin, Italy; francescopetrillo09@gmail.com (F.P.); mreibaldi@libero.it (M.R.); 4 National Biodiversity Future Center (NBFC), 90133 Palermo, Italy; 5 Center for Studies on Bioinspired Agro-Environmental Technology (BAT Center), 80055 Portici, Italy

**Keywords:** polyunsaturated fatty acids, resolvin, mixed biofilm, *Candida parapsilosis*, *Staphylococcus aureus*

## Abstract

The fungal species *Candida parapsilosis* and the bacterial species *Staphylococcus aureus* may be responsible for hospital-acquired infections in patients undergoing invasive medical interventions or surgical procedures and often coinfect critically ill patients in complicating polymicrobial biofilms. The efficacy of the re-purposing therapy has recently been reported as an alternative to be used. PUFAs (polyunsaturated fatty acids) may be used alone or in combination with currently available traditional antimicrobials to prevent and manage various infections overcoming antimicrobial resistance. The objectives of the study were to evaluate the effects of Resolvin D1 (RvD1) as an antimicrobial on *S. aureus* and *C. parapsilosis*, as well as the activity against the mixed biofilm of the same two species. Microdilution assays and time–kill growth curves revealed bacterial and fungal inhibition at minimum concentration values between 5 and 10 μg mL^−1^. In single-species structures, an inhibition of 55% and 42% was reported for *S. aureus* and *C. parapsilosis*, respectively. Moreover, RvD1 demonstrated an eradication capacity of 60% and 80% for single- and mixed-species biofilms, respectively. In association with the inhibition activity, a downregulation of genes involved in biofilm formation as well as ROS accumulation was observed. Eradication capability was confirmed also on mature mixed biofilm grown on silicone platelets as shown by scanning electron microscopy (SEM). In conclusion, RvD1 was efficient against mono and polymicrobial biofilms in vitro, being a promising alternative for the treatment of mixed bacterial/fungal infections.

## 1. Introduction

In nature, most microorganisms are associated with abiotic or biotic surfaces forming multispecies consortia called biofilms. Such microbial communities are enclosed in a self-produced polymeric matrix, mainly composed of polysaccharides, lipids, proteins, and extracellular DNA. Microorganisms within biofilms are known to be refractory to antimicrobials and the host immune response. Many human diseases are affected by the colonization of multispecies communities within the human body, especially of immunocompromised individuals, negatively affecting treatments and increasing health risks [1]. The prevalence of biofilms in infection-related diseases is over 75%. The presence of biofilms greatly increases the antimicrobial resistance of microorganisms up to 10–1000 times compared to their planktonic counterparts [2]. Once a biofilm has formed, it is extremely difficult to eradicate with the use of generic antimicrobials. Medical devices, such as central venous catheters (CVCs), are often contaminated and colonized with biofilms that could lead to biofilm-related infections. In particular, polymicrobial biofilms have a significant impact on the pathogenesis of infectious microorganisms [3].

In a polymicrobial biofilm where multiple microbial species are closely associated, mutually beneficial interactions can develop. Polymicrobial biofilms found in almost all niches of the human body such as the oral cavity and gastrointestinal and urogenital tracts have been extensively studied although little is known about the behavior of mixed microorganism communities, particularly fungal-bacterial biofilms [2]. The formation of polymicrobial biofilms has been linked to a variety of human diseases, including oral cavity infection, otitis media, chronic lung infection, burn wounds, urinary tract infection, and medical device-related infection [4,5,6,7,8]. Several studies show that polymicrobial interactions result in increased tolerance to antimicrobial agents or in a variety of other environmental stress conditions and evade host immune responses. Consequently, the failure of antimicrobial treatment against biofilm-forming microbial pathogens is increasing and it is necessary to develop alternative strategies to combat polymicrobial diseases [9]. Therefore, it is crucial to explore new antimicrobial agents to combat biofilm formation.

An increasing number of reports describe the formation of polymicrobial biofilms between *Candida* spp., especially *C. albicans*, and various bacterial species [10]. *Candida* spp. and *Staphylococcus aureus* are the main opportunistic pathogens in nosocomial infections and are classified among the top three bloodstream pathogens. They are reported to cause severe morbidity and mortality in hospitalized patients, being co-isolated in biofilms from various mucosal surfaces including [11]. Bloodstream infection (BSI) is a serious clinical condition that affected 8.3% of intensive care unit (ICU) patients in 2017 with 37% of the cases being catheter-related. The most frequently isolated microorganisms in BSI episodes were coagulase-negative *Staphylococci* spp., *Enterococcus* spp., *Klebsiella* spp., and *Staphylococcus aureus*, and the yeast *Candida parapsilosis* is the most common source of BSI among *Candida* spp. [12].

*S. aureus* is a major cause of nosocomial and community-acquired infections with a diverse range of infections from acute infections bacteremia and skin abscesses, to chronic infections, such as osteomyelitis and endocarditis [13]. These infections, particularly biofilm-related infections are recalcitrant to treatment with antimicrobials and host defense molecules. In clinical settings, *S. aureus* forms biofilms resistant to antibiotics and innate host defense mechanisms on the surfaces of the catheters (intravenous, urinary, dialysis catheters) and implanted medical devices (joint prostheses, pacemakers, etc.). It is also reported that *S. aureus* can form polymicrobial biofilms with *Candida* spp. both in vitro and in vivo [14,15,16]. It is estimated that 27% of nosocomial *C. albicans* bloodstream infections are polymicrobial with *S. aureus* [17].

The frequency of Invasive infections by *non-albicans Candida* species, especially *Candida parapsilosis*, has increased over the past few decades with *C. parapsilosis* the second most commonly isolated species from bloodstream infections [18]. *C. parapsilosis*, part of the human skin microbiota, is often isolated from the hands of hospital workers, and is able to produce biofilms on central venous catheters (CVCs) and other medical implants such as catheter-related infections. The ability to form biofilms has been considered to be an important, if not the major, virulence factor of *C. parapsilosis*, although has not been considered to form true hyphae like *C. albicans*. The ability to form biofilm on different surfaces can be simple or mixed biofilm [19,20]. Medical interests are turning to investigate the links between the prokaryotic pathogen *S. aureus* and the eukaryotic pathogen *C. parapsilosis* during the formation of polymicrobial biofilms, but above all to explore new antimicrobials because normal treatments may be ineffective, substandard, or fail under these conditions [21].

Recently, drug repurposing is attracting considerable interest as an alternative approach to discovering new potential antimicrobials. Since these drugs are approved by the Food and Drug Administration (FDA), information on their pharmacological characteristics (chemical stability, toxicity, dosage, action kinetics, etc.) is readily available, reducing the time and economic costs required to evaluate new therapeutic applications [22,23,24].

Omega-3 polyunsaturated fatty acids (PUFAs), especially eicosapentaenoic acid and docosahexaenoic acid (DHA), are dietary supplements naturally present in fish and spices, and have known favorable effects on cardiovascular outcomes [25]. In particular, Resolvin D1 (RvD1), derived from DHA, has been reported to show potent anti-inflammatory effects in several animal models [26]. RvD1 belongs to the class of so-called pro-resolving lipid mediators, such as lipoxins, resolvins, maresins, and protectins, tested for their ability to combat local and systemic inflammatory conditions [27]. Petrillo et al. 2020 [28] showed that RvD1 ameliorated the LPS-induced keratitis in mice and reduced the corneal damage affecting a pattern of mediators proper to inflammation and tissue repair.

Some marine PUFAs such as stearidonic acid (C18:4 n-3), eicosapentaenoic acid (C20:5 n-3), and docosapentaenoic acid (C22:5 n-3) were found to inhibit *C. albicans* and *C. dubliniensis* biofilm biomass production in vitro. The authors speculated that these effects might be due to oxidative stress, leading to apoptosis [29].

Following the above-reported evidence, in this study, we propose the use of RvD1 to *S. aureus*–*C. parapsilosis* mixed biofilms. Aiming at this, after assessing the antimicrobial activity of RvD1 on the planktonic cells of the examined species, we examined the preventive and eradicating effects of RvD1 in an in vitro model. Further, we investigated the effect of RvD1 during the inhibition of the dual-species biofilm formation, on both the expression levels of some biofilm-related genes (*ERG11*, *EFG1*, *ALS3* for *C. parapsilosis*, and *icaA*, *icaD*, and *bap* for *S. aureus*) and the ROS production. Also, the inhibitory effect of RvD1 was studied on the adhesion of the two microorganisms on HaCaT epithelial cells.

Silicone is widely used in clinics for catheters and medical devices due to its high biocompatibility and versatility [30], though it becomes rapidly colonized by microorganisms forming biofilms which increase the risk of associated infections. Therefore, in this work, we also assessed the eradication ability of RvD1 on the dual-species *S. aureus*–*C. parapsilosis* biofilm which had been established on a silicone surface.

## 2. Results

### 2.1. Effect of RvD1 on Planktonic Cell Growth

**Figure 1:**
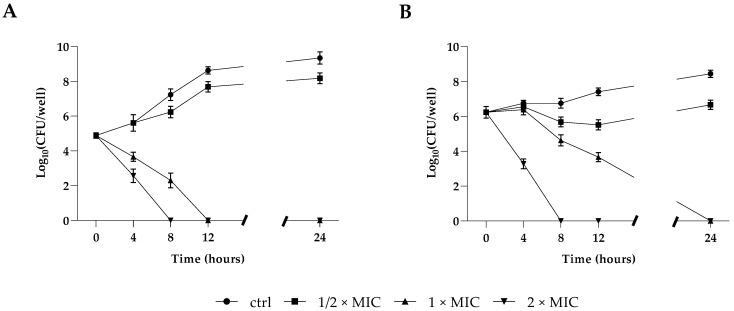
Killing kinetics of *S. aureus* (**A**) and *C. parapsilosis* (**B**) cultured in the presence of 1/2 × MIC, 1 × MIC, and 2 × MIC concentrations of RvD1 over a period of 24 h. Data reported are the mean of three independent experiments ± SD.

The antifungal potential of the Resolvin D1 (RvD1) against planktonic growth of the two microorganisms was determined and expressed as MIC (minimum inhibitory concentration) values. As shown in Table 1, the MICs and MBCs (minimum bactericidal concentration) of *S. aureus* were 5 and 15 μg mL^−1^, respectively, whereas MICs and MFCs (minimum fungicidal concentration) for *C. parapsilosis* were 10 and 25 μg mL^−1^, respectively, showing in both cases a bactericidal/fungicidal activity. In accordance with our above results, the time–kill growth curve showed that RvD1 significantly inhibited *S. aureus* (Figure 1, panel A) and *C. parapsilosis* (Figure 1, panel B) planktonic cell growth in a dose-dependent manner within 12/24 h of treatment. In fact, the treatment with RvD1 at 1/2 × MIC concentration did not induce significant deflections of microbial growth curves at 24 h compared to control samples. Conversely, the treatment with 1 × MIC showed a reduction in bacterial load already observed after 4 h of incubation in the case of *S. aureus* culture, with a complete bactericidal action after 12 h. Moreover, for *C. parapsilosis*, the treatment with 1 × MIC RvD1 showed a decrease relative to the initial load after 8 h of treatment, with a full fungicidal action after 24 h. In both cases, the ratios MBC or MFC/MIC were lower than 4.0, indicating a biocidal activity of RvD1.

### 2.2. Mono-Microbial or Poly-Microbial Biofilms of S. aureus and C. parapsilosis

**Figure 2:**
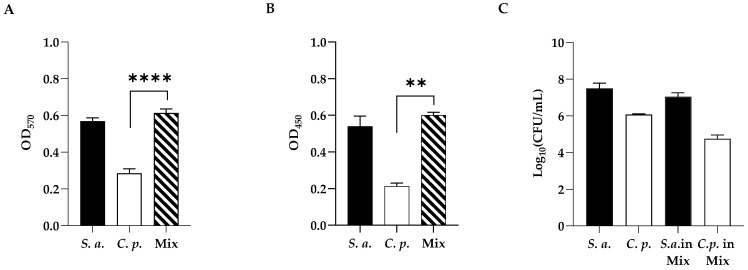
(**A**) Standard optical density biofilm CV assay of single-species and dual-species biofilms. (**B**) XTT metabolic activity assay of single-species and dual-species biofilms. (**C**) Cell enumeration in dual-species biofilms. Statistical significance: ** = *p* < 0.01; **** = *p* < 0.0001 (Dunnett’s test). Data reported are the mean of three independent experiments ± SD.

The development of mono-microbial or poly-microbial biofilms of *S. aureus* and *C. parapsilosis* were confirmed by both crystal violet (CV) (Figure 2, panel A) and XTT (Figure 2, panel B) staining to determine the total biomass and the metabolic activity of single and dual-species biofilms, respectively. As shown in Figure 2 (panel A), single strains showed strong biofilm production in the case of the bacterium, and a moderate one for the fungus, while the two species showed a strong formation ability to form a dual-species biofilm in vitro. The metabolic activity of biofilm cells was evaluated by the XTT assay (Figure 2, panel B), and as well as the biofilm biomass, the metabolic activity was also found to increase in the dual-species biofilm.

As shown in Figure 2 (panel C), to better characterize the mono- and polymicrobial biofilms, viable cell numbers of both *C. parapsilosis* and *S. aureus* alone or co-cultured, were enumerated by cell count. As expected, the highest count was recorded for *S. aureus* compared to *C. parapsilosis* single biofilms (6 Log and 8 Log, respectively), showing that in the mixed biofilm *S. aureus* was dominating. In dual-species biofilms, as reported in our previous study [31], fungi and bacteria had a different distribution with the bacterium predominating on the upper layer of the biofilm with a preferential adherence to hyphae.

### 2.3. Inhibition/Eradication of Mono- and Poly-Microbial Biofilms in 96-Well Microplate

**Figure 3:**
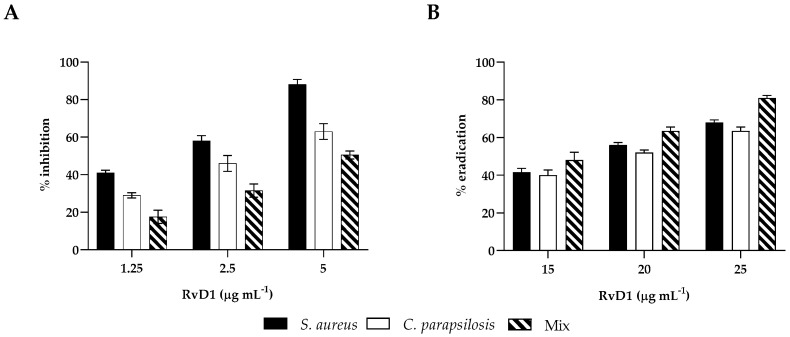
Biofilm inhibitory and eradication effect of RvD1 towards single- and mixed-species biofilms. (**A**) Inhibition effect of RvD1 on mono-microbial and poly-microbial biofilm formations in 96-well microplates. Single and mixed species cells were co-incubated with various concentrations (1.25, 2.5, and 5 µg mL^−1^) of RvD1 for 24 h; and their biofilm production was compared to that of cells incubated without RvD1 (CV assay). (**B**) Eradication effect of RvD1 on mono- and poly-microbial mature biofilms in 96-well microtiter plates. Mature biofilms were treated with various concentrations (15, 20, and 25 µg mL^−1^) of RvD1 for 24 h. Values obtained are given as the percentage of biofilm formation or eradication. The results shown represent the means and standard deviations (error bars) of three independent experiments.

The CV assay was used to test the effects of RvD1 at sub-MIC concentrations on monomicrobial and poly-microbial biofilms of *S. aureus* and *C. parapsilosis*. Biofilm formation was significantly inhibited by RvD1. Indeed, both *S. aureus* and *C. parapsilosis* (Figure 3, panel A) biofilms demonstrated similar patterns of inhibition in a concentration-dependent manner. At 2.5 µg mL^−1^ RvD1, biofilm formation by *S. aureus* and *C. parapsilosis* was inhibited by 55% and 42%, respectively. Instead, a higher dose was needed to inhibit the formation of the polymicrobial biofilm since 50% of inhibition was observed at 5 µg mL^−1^ RvD1. Considering the eradication activity, no effect of RvD1 was seen on either the single or dual-species biofilm at sub-MIC concentrations. It was necessary to reach a concentration of 25 µg mL^−1^ to have 60% and 80% eradication of the mono- and mixed biofilms, respectively (Figure 3, panel B).

### 2.4. Reactive Oxygen Species Assay

**Figure 4:**
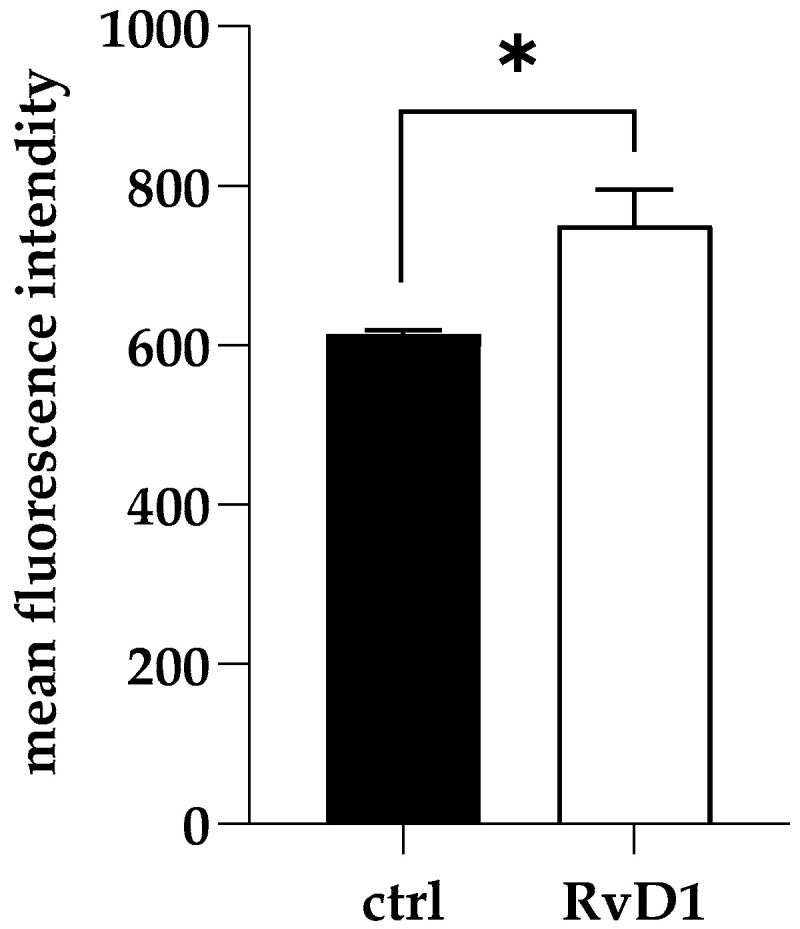
Accumulation of ROS in *S. aureus* and *C. parapsilosis* mixed biofilms following RvD1 supplementation. Values are averages of triplicate experiments and error bars indicate the standard deviation. * = significantly different from control (*p* < 0.01).

To explore the early damage caused by RvD1 (2.5 µg mL^−1^) on dual-species biofilm during inhibition, ROS production was measured. As shown in Figure 4, RvD1 induced significant production of intracellular ROS compared to the control. 

### 2.5. RvD1 Inhibited the Expression of Biofilm Formation-Related Genes

**Figure 5:**
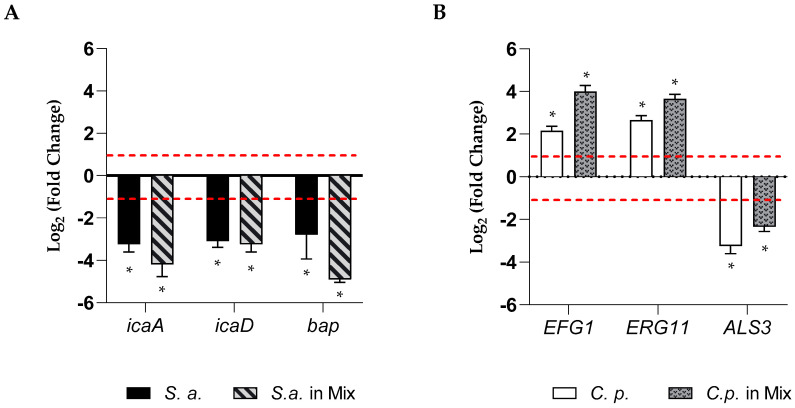
Relative fold-change in gene expression of *S. aureus* and *C. parapsilosis*. (**A**) The chart represents changes in expression of *bap*, *icaA*, and *icaD* genes in mono and poly-microbial biofilms of *S. aureus*, in the presence of sub-inhibitory concertation of RvD1 (2.5 µg mL^−1^) compared to untreated samples and normalized to the value of *16S rRNA*. (**B**) The chart represents relative changes in expression of the *ALS3*, *EFG1*, and *ERG11* genes in mono and poly-microbial biofilms of *C. parapsilosis* during inhibition with 2.5 µg mL^−1^ RvD1 compared to untreated *C. parapsilosis* as a control and normalized to the value of *actin*. Asterisk marked fold changes significantly different from 1 (*p*-value < 0.05). Red broken lines indicate fold change thresholds of 2 and 0.5, respectively.

To elucidate the possible effects involved in the anti-biofilm action of RvD1, we quantified the expression of genes related to biofilm formation for the two microorganisms. The expression of three genes for each microorganism in single or dual-biofilms during inhibition treatment in the presence of a sub-inhibitory concentration of RvD1 (2.5 µg mL^−1^) was detected. Results are summarized in Figure 5 (panels A, B). Data suggested that the expression of the three genes of *S. aureus*, *icaD*, *icaA* (both involved in adhesion), and *bap* (codifying for a biofilm-associated protein) (Figure 5, panel A) were considerably downregulated, showing a value of Log 2 fold change of −3.2, −3.1, and −2.8, respectively, in mono-microbial biofilm, and −4.2, −3.2, and –4.9, respectively, in poly-microbial biofilm, showing that they were significantly affected by RvD1 treatment. In the presence of a sub-inhibitory concentration of RvD1, the expression of the *ERG11* and *EFG1* genes of *C. parapsilosis* were slightly increased in both mono- and dual-species biofilms (Figure 5, panel B), while *ALS3* was significantly downregulated by RvD1 treatment.

### 2.6. Eradication of Polymicrobial Biofilms on Silicone

**Figure 6:**
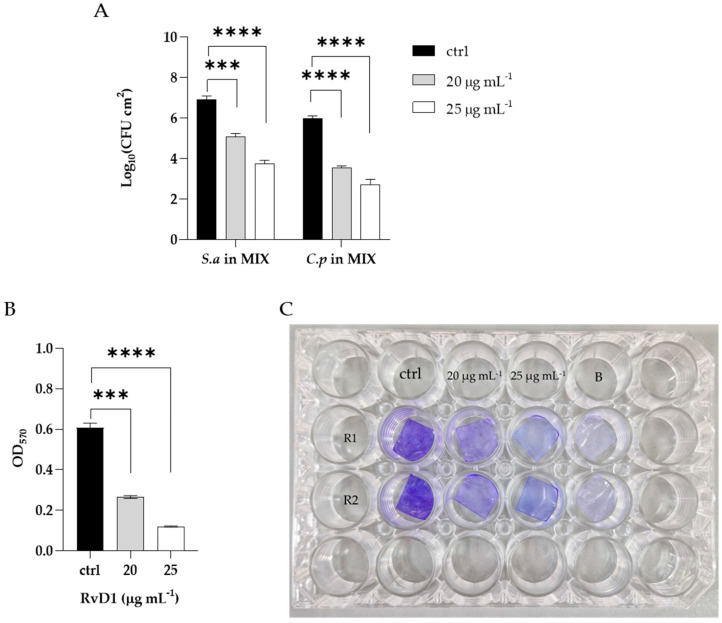
Eradication of mixed biofilm by RvD1 on medical grade silicone surface. Polymicrobial biofilms were grown on medical grade silicone surface for 72 h and treated with two concentrations (20; 25 µg mL^−1^) of RvD1. (**A**) CFUs of each microorganism in the mixed biofilm treated compared with untreated; (**B**) total biomass detected by CV of the mixed biofilm treated with two concentrations of RvD1. (**C**) Crystal violet staining of silicone platelets colonized by *S. aureus*–*C. parapsilosis* biofilm and treated with 20 and 25 µg mL^−1^ RvD1. ctrl = control (untreated), B = blank (without cultures), R1 = replies 1, R2 = replies 2; *** = *p* < 0.001; **** = *p* < 0.0001 (Dunnett’s test).

**Figure 7:**
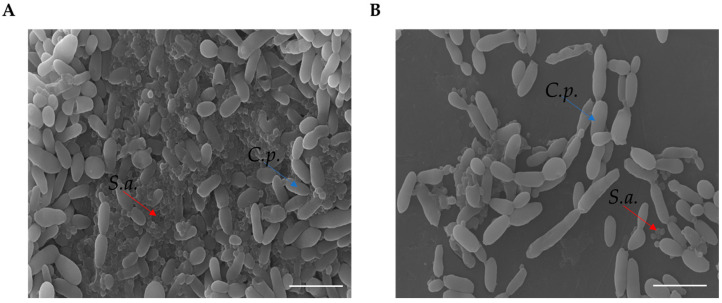
SEM images of polymicrobial biofilm formations on medical grade silicone surface with media supplemented without (**A**) or with RvD1 (**B**). Magnification, ×1000. Bar corresponds to 10 µm. Red arrows indicate *S. aureus* (S.a.), blue arrows indicate *C. parapsilosis* (C.p).

In order to determine the eradication ability of RvD1 on the polymicrobial biofilm formation on silicone, mixed species biofilms were allowed to establish on the surface of medical silicone for 72 h and treated for 24 h with RvD1 (20 and 25 µg mL^−1^). Quantization of the residual biofilm remaining attached to the platelets with CV (Figure 6, panel B) showed that the polymicrobial biofilm was eradicated in a similar manner to that grown in 96-well microplate following a dose-dependent manner. The results revealed (Figure 6, panel A) a decrease of 2 Log compared to the control at 20 µg mL^−1^ RvD1 for both *S. aureus* and *C. parapsilosis*, and a reduction of 4 Log and 3 Log compared to the control for the bacterium and fungus, respectively with the treatment at higher dose (25 µg mL^−1^). The eradication consequence of RvD1 on the architecture of mixed-species biofilms was investigated by scanning electron microscopy (SEM). As shown in Figure 7, the SEM image of the untreated *S. aureus*/*C. parapsilosis* mixed biofilm was highly dense and aggregated (panel A). On the contrary, the treated sample showed a reduced-adhered population in comparison to the untreated biofilm cells (panel B), proving the good eradication ability of RvD1 also on silicone surfaces.

### 2.7. Cytotoxicity and Protective Role of RvD1 against Microbial Adhesion 

**Figure 8:**
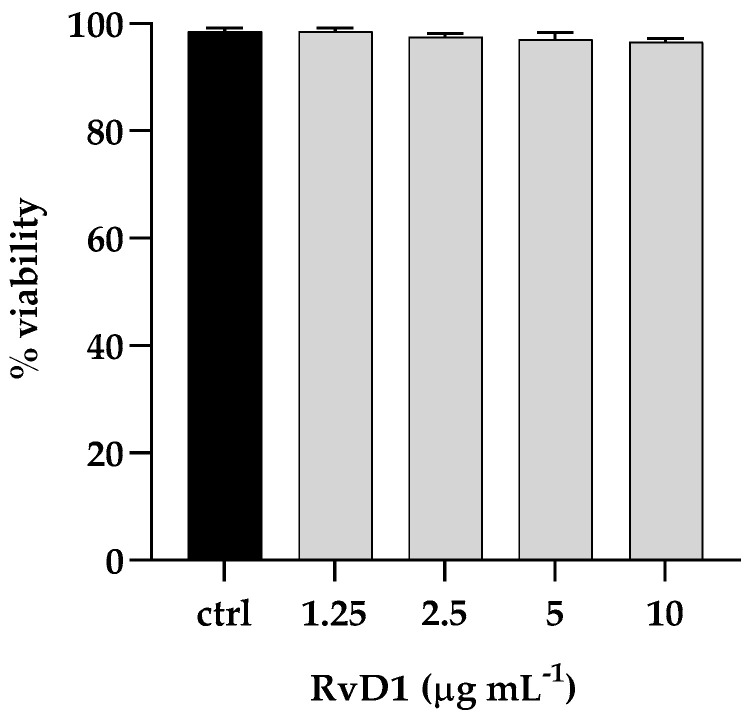
Cytotoxic effect (%) of RvD1 on the HaCaT cell line after 24 h of treatment with concentrations ranging from 1.25 to 10 μg mL^−1^. Data represent mean ± standard deviation (SD) of three independent experiments.

**Figure 9:**
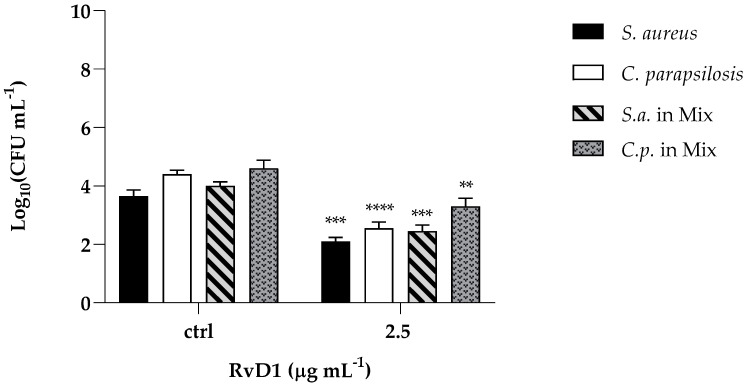
Effect of RvD1 on the adhesion of *S. aureus* and *C. parapsilosis* mono- and dual-specie culture to HaCaT cells expressed by CFU mL^−1^. The results were obtained from two independent experiments: ** = *p* < 0.01; *** = *p* < 0.001, **** = *p* < 0.0001 (Tukey’s test).

The cytotoxic activity of RvD1 was evaluated on human spontaneously immortalized HaCat keratinocytes using the colorimetric assay, 3-(4,5-dimethylthiazol-2-yl)-2,5-diphenyl-2H-tetrazolium bromide (MTT). Cells were treated with different concentrations of the compound (ranging from 1.25 to 10 μg mL^−1^) and then subjected to the MTT assay. The cytotoxic activity was expressed as a percentage of viability relative to the viability of untreated cells. No cytotoxicity was pointed out by the MTT assay, and the survival of treated cells was comparable to that of control cells (Figure 8). Further, to assess the anti-infective nature of RvD1 we determined *C. parapsilosis* and *S. aureus* adhesion ability in presence of the drug. As reported in Figure 9, the compound at concentration of 2.5 μg mL^−1^ affect both *S. aureus* and *C. albicans* adherence to HaCaT cells, as demonstrated by the significant reduction of adherent cells of both species, when inoculated alone or together. Overall, these data suggest that RvD1 could be a potential drug to prevent mixed fungal/bacterial infections of epithelial cells.

## 3. Discussion

Bacterial/fungal biofilms, namely polymicrobial communities, are considered one of the leading conditions predisposing to invasive human infections also related to hospitalization of clinical complexity and to many co-morbidities, such as cancer, chronic diseases, neutropenia, transplants, and mucositis [32]. These communities represent a big threat because extended stay in intensive care medicine, use of mechanical supports, parenteral nutrition and repeated use of drugs that favor overgrowth and invasion of pathogens, are relevant factors associated with the difficulty of inhibition and eradication of the biofilms produced by them. As reported in the literature, not only is the possibility that antibacterial and antifungal drugs solving polymicrobial infections limited (70% treatment failure), but also, they mutually increase antimicrobial resistance [33]. The development of resistance towards antimicrobial agents already in use makes it necessary to search for novel antimicrobials. Fatty acids have been reported to exhibit a high degree of specificity towards biofilms [34,35].

Previous studies have suggested that both short- and long-chain PUFA are potent endogenous antimicrobials against a variety of Gram-positive and Gram-negative bacteria producing ultrastructural damage to the bacterial plasmatic membrane, and also exhibit anti-fungal, anti-viral, and anti-parasitic activities [36]. Abdullatif et al., 2022 [37] reported that resolving E1 (RvE1) derived from polyunsaturated eicosapentaenoic acid showed significant antimicrobial activity against *A. actinomycetemcomitans* bacteria on the subgingival microbiota of both rats and rabbits with experimental periodontitis. Wu et al., 2023 [38] demonstrated the capacity of RvD1 to improve the prognosis of keratitis caused by *Pseudomonas aeruginosa* not only by dampening the inflammatory response but also by reducing the bacterial burden in PA-infected corneas with a complementary therapy together with gatifloxacin eye drops than those treated with gatifloxacin alone. Similarly, previous studies have suggested that RvD1, in addition to ciprofloxacin, was more effective at reducing blood bacterial titers in murine peritoneal *E. coli* infections than ciprofloxacin alone and can also amplify the action of vancomycin in *Staphylococcus aureus* skin infections [39]. 

In this study, we demonstrated the strong anti-biofilm activity of a repurposed drug, Resolvin D1, derived from docosahexaenoic acid, on both *S. aureus* and *C. parapsilosis* single and dual-species biofilms. RvD1 could also significantly eradicate dual mature biofilms on silicone surfaces. RvD1 showed significant anti-*S. aureus* effects but moderate anti-*C. parapsilosis* effects. We report that the MIC and MBC of RvD1 against *S. aureus* were 5 and 15 μg mL^−1^, respectively, while 10 and 25 μg mL^−1^ for *C. parapsilosis*, respectively, showing a bacteriostatic/fungistatic activity. After 12 and 24 h, at a 1 × MIC RvD1 concentration, we observed the complete killing of *S. aureus* and *C. parapsilosis*, respectively.

A crucial aspect in preventing mono or dual-species biofilm formation concerns the early stages of pathogens adhesion, when the interaction with the abiotic surface is still reversible, becoming irreversible when the biofilm is formed. Here, we assessed the good preventive ability of RvD1 toward both mono- and dual-species biofilms. RvD1 was able to interfere with the expression of biofilm-associated genes, such as *bap*, *icaD*, and *icaA* for *S. aureus* and *ALS3* adhesins of *C. parapsilosis*, demonstrating a specific ability to interfere with the early stages of adhesion. We also measured ROS accumulation in the biofilm-forming cells of *S. aureus* and *C. parapsilosis*, by exposing such cells to RvD1 during the early phase of the mixed biofilm formation to ascertain the potential ability of RvD1 to inhibit such formation (adhesion and subsequent surface colonization). Our results corroborated the inhibitory effect of RvD1 at a sub-MIC concentration on the mixed biofilm formation at sub-MIC concentration. It is widely known that ROS production can damage cell constituents (proteins, lipids, nucleic acids), inducing structural changes that lead to a loss of cell functions up to cell apoptosis. Many antimicrobial compounds, either synthetic or of natural origin, induce a common oxidative cellular damage, suggesting that at least part of their effect depends on ROS production. In particular, an increase in ROS is ascertained in the case of PUFAs, which are reported to cause ultrastructural differences, attributable to increased oxidative stress, between treated and untreated *Candida* cells [29].

Polymicrobial interaction causes robust biofilm formation in the host as well as on the surface of medical devices, which become a source of infection. Here we showed that RvD1 can disrupt preformed or mature dual-species biofilms grown on two different surfaces: polystyrene and silicone. The higher concentrations (>MIC value) required for both conditions pre-formed and mature biofilm eradication, can be attributed to the recalcitrant nature of the established mature biofilm due to the presence of an exopolymeric matrix which establishes during the development phase of biofilm formation which acts as a barrier to the penetration of drugs. The non-cytotoxic effect at the doses tested in mammalian cells and the ability to avoid adherence to them, adhesion is the first step in the interaction of pathogens with the host and in the subsequent possible biofilm formation, corroborate the promising possibility of using RvD1 as new compound for biofilms treatment.

In conclusion, our results showed a strong effect of RvD1 against monomicrobial and polymicrobial biofilms of *S. aureus* and *C. parapsilosis*, although further studies in vivo are required to better understand and confirm RvD1’s activity promises, for it be a practical and simple new agent for biofilm treatment and if it is also possible that bioactive lipids may be beneficial to overcome drug resistance. 

## 4. Materials and Methods

### 4.1. Strains, Media, and Reagents

The reference strains *C. parapsilosis* DSM 5784 and *S. aureus* ATCC 6538 used in this study were kept in our laboratory on Yeast Peptone Dextrose Agar (YPD, Sigma-Aldrich, Wien, Austria) and Tryptone Soya Agar (TSA, OXOID, Basingstoke, UK) and were cultured in Tryptone Soya Broth (TSB) supplemented or not with 1% *w*/*v* glucose, respectively. TSB supplemented with 1% glucose was used for the dual-species biofilm growth as reported previously [40]. RvD1 with ≥95% of purity was purchased from Cayman Chemical (Ann Arbor, MI, USA) dissolved in ethanol at a final concentration of 50 mg mL^−1^.

### 4.2. Determination of the Minimum Inhibitory Concentration and Minimum Fungicidal/Bactericidal Concentration 

The MIC and MBC/MFC were assessed for RvD1 through the plate microdilution assay, according to the Clinical and Laboratory Standards Institute (CLSI) guidelines CLSI M27-A3 and M07-A9 [41,42]. Briefly, 100 μL of fungal or bacterial culture was diluted to a final concentration of 1 × 10^6^ cells mL^−1^ in TSB with or without glucose, for *C. parapsilosis* and *S. aureus*, respectively, and added into each well of a 96-well-microplate with RvD1 ranging from 0.5 to 50 μg mL^−1^. Microbial cultures were incubated at 37 °C for 24 h and microbial growth was determined at 590 nm wavelength with a microplate reader (SYNERGYH4 BioTek). MICs were determined as the lowest drug concentration that produced ≥90% inhibition of growth relative to growth control. The MBC/MFC was the smallest drug concentration that inhibited microbial growth on the agar plates. The MFC/MIC ratio was calculated to determine whether the substance had biostatic (MFC/MIC ≥ 4) or biocidal (MFC/MIC < 4) activity, as reported previously [43].

### 4.3. Time Killing Assay

Time killing curves were analyzed to monitor bacterial/fungal growth in response to increasing concentrations of RvD1 over time. Concentrations corresponding to 1/2 × MIC, 1 × MIC, 2 × MIC, were added in flasks containing the proper medium with an initial microbial inoculum of 1 × 10^6^ CFU mL^−1^ and incubated at 37 °C. Untreated microorganisms were used as negative controls. Aliquots of 100 μL of suspensions were collected from the flasks at 0, 1, 3, 6, and 20 h, serially diluted in phosphate-buffered saline (PBS) and plated on TSA or TSA plus 1% glucose for *S. aureus* and *C. parapsilosis*, respectively, and then incubated at 37 °C for 24 h. After incubation, colonies were counted and CFU mL^−1^ values were calculated.

### 4.4. Simple and Mixed Biofilm Formation and Quantification

First, the biofilm production ability of the two strains alone or together used in this study was evaluated using the method described in our previous study [44] with some modifications. Briefly, overnight suspensions of *C. parapsilosis* and *S. aureus* were standardized to 10^6^ cells mL^−1^ and transferred to a 96-well plate. Dual-species biofilms were formed in flat-bottomed microtiter plates by adding the microbial suspensions 1:1 ratio spectrophotometrically adjusted to 10^6^ cells mL^−1^ for *C. parapsilosis* + 10^6^ cells mL^−1^ for *S. aureus* in the medium. After incubation of the microwell plate for 24 h at 37 °C, the supernatant medium was removed, and the underlying adherent cells were washed three times with PBS. Residual biofilm total biomass was quantified by the cCV assay [45,46]. Biofilms were fixed in methanol for 15 min, stained with 0.2% (*v*/*v*) CV for 15 min, and then wells were washed with sterile water and dried for 30 min at 37 °C. Biofilms were de-stained by treatment with 300 μL 33% (*v*/*v*) glacial acetic acid for 15 min and measured at OD_570_ nm using a microtiter plate reader. Results were interpreted according to Stepanović [45] as follows: OD ≤ ODc (OD cut-off) non-biofilm producer (NBP); ODc < OD ≤ 2 × ODc weak biofilm producer (WBP); 2 × ODc < OD ≤ 4 × ODc moderate biofilm producer (MBP); 4 × ODc < OD strong biofilm producer (SBP). At least three independent experiments in triplicate were conducted. Biofilm viability was detected by evaluating the metabolic activity of the biofilms with the 2,3-bis(2-methoxy-4-nitro-5-sulfophenyl)-5-[(phenylamino) carbonyl]-2H-tetrazolium hydroxide (XTT; Sigma-Aldrich) reduction method as previously described [47] and according to the manufacturer’s instructions. The absorbance values were measured at 492 nm using a microtiter plate reader. Counting of colony-forming units (CFUs) was performed to evaluate the number of cultivatable cells. Biofilms cells scraped from the wells were homogenized and serially diluted and plated into Petri dishes containing TSA (with amphotericin B, 1 μg mL^−1^) or Rose Bengal (with chloramphenicol, 1 μg mL^−1^) agar, to distinguish *S. aureus* and *C. parapsilosis*, respectively, and hence incubated at 37 °C for 24/48 h before counting.

### 4.5. Antibiofilm Activity

To test the inhibition activity of RvD1, mono- and dual-species biofilms were prepared as described in paragraph 4.4 but added to the microbial cell suspensions in the culture medium with different sub-MIC concentrations of RvD1, ranging from 1.25 to 5 μg mL^−1^. After incubation at 37 °C for 24 h, the residual biofilm was detected by performing the CV staining, as reported above.

The disruption (eradication) efficiency of RvD1 towards mature biofilms of the two species alone or together was carried out by allowing them to form the biofilms, and then adding different concentrations of RvD1 at sub-MIC concentrations (15; 20; 25 μg mL^−1^) for other 24 h at 37 °C. Then, the planktonic cells were removed by PBS washing, and the residual biofilm cells were quantified by CV staining, as reported above.

### 4.6. Measurement of Reactive Oxygen Species (ROS)

Accumulation of intracellular ROS production was measured using the fluorescent dye, 2′,7-dichlorofluorescin diacetate (DCFHDA) (Sigma Aldrich, Agilent Technologies, Winooski, VT 05404, USA). Briefly, biofilms were prepared as described above in 96-well microtiter plates, by incubating mixed culture with 2.5 μg mL^−1^ of RvD1 in micro-well plates at 37 °C for 24 h to allow biofilm formation. Then, biofilms were washed twice with sterile PBS to remove non-adherent cells, treated with a volume of 10 mM DCFHDA, and incubated at 37 °C in the dark. Fluorescence intensities (excitation and emission of 488 and 540 nm, respectively) were quantified by a microtiter plate reader (Synergy H4; BioTek Instruments, Inc., Winooski, VT, USA).

### 4.7. Detection of Biofilm-Associated Genes

The following biofilm-associated genes *ERG11*, *EFG1*, *ALS3* for *C. parapsilosis*, *icaD*, *icaA*, and *bap* for *S. aureus* were amplified by PCR using specific primers (Table S1) to evaluate the RvD1 effect during inhibition of both mono- and dual-species biofilm formation. Briefly, the biofilm mass was scraped, and washed in PBS and total RNA was isolated using Direct-zolTM RNA Miniprep Plus Kit (ZYMO RESEARCH). Then, 1 μg of RNA was reverse-transcribed to cDNA (Bio-Rad, Milan, Italy) and analyzed by quantitative PCR run in AriaMx Real-Time PCR instrument (Agilent Technologies, Inc., Milan, Italy) according to the manufacturer’s instructions. PCR System used: 95 °C for 10 min, one cycle for cDNA denaturation; 95 °C for 15 s and 60 °C for 1 min, 40 cycles for amplification; 95 °C for 15 s, one cycle for final elongation; and one cycle for melting curve analysis (from 60 °C to 95 °C) to verify the presence of a single product. The expression of each gene was analyzed and normalized against *16S rRNA* and *ACT1* gene for *S. aureus* and *C. parapsilosis*, respectively, using REST software (Relative Expression Software Tool, Weihenstephan, Germany, version 1.9.12) based on the Pfaffl method [48,49]. 

### 4.8. Growth and Treatment of Long-Term Dual-Species Biofilms on Medical Silicone Surface 

The long-term (72 h) biofilm was formed as previously described [50]. In brief, medical-grade silicone platelets (0.5 mm thick) were placed into a 24-well microplate (Novotema S.p.a. Villongo, BE, Italy), and incubated in FBS for 24 h. Then, overnight *C. parapsilosis* and *S. aureus* cultures were diluted to 1 × 10^6^ cells mL^−1^ each in TSB-1% glucose mixed and added to the wells at 37 °C for 90 min (adhesion phase). Non-adherent cells were removed, and the microplate was incubated under shaking with TSB-1% glucose containing different concentrations of RvD1 for 72 h at 37 °C renewing medium every 24 h. For controls, platelets were processed in an identical way, except that no microorganism was added. To determine the number of adherent cells of the biofilm after treatment, the platelets were washed in PBS to remove non-adherent cells and carefully scraped, and then the CFU assay was performed. The number of viable cells was reported as Log CFU cm^−2^ of silicone surface.

### 4.9. SEM Analyses

To perform scanning electron microscopy (SEM) observation of mixed biofilms grown on silicone and treated with RvD1 (25 µg mL^−1^), the biofilms were washed with PBS, fixed overnight with 3% *v*/*v* glutaraldehyde at 4 °C. After fixation in 1% *p*/*v* aqueous solution of osmium for 90 min at room temperature, samples were dehydrated in a series of graded alcohols, dried to the critical drying point, and finally coated with gold, as reported previously [51]. Specimens were evaluated with a scanning electron microscope (QUANTA 200 ESEM FEI Europe Company, Eindhoven, The Netherlands).

### 4.10. Cytotoxicity Assays

HaCaT cells were used to evaluate the cytotoxicity of RvD1 by MTT assay. The cells were grown in Dulbecco’s modified Eagle medium (DMEM, Sigma Aldrich), supplemented with 1% penicillin-streptomycin, 1% L-glutamine, and 10% Fetal Bovine Serum (Sigma Aldrich), at 37 °C with 5% CO_2_ in a humid environment. A density of 2 × 10^4^ cells/well was seeded in 96-well plates and incubated for 24 h. A concentration ranging between 1 and 10 μg mL^−1^ of RvD1 was used for cell treatment. Untreated cells were used as a negative control. After 24 h of treatment, MTT solution was added to each well for 4 h at 37 °C. The solubilization of the formazan crystals was achieved by adding DMSO and the viability was recorded at OD at 570 nm, using a microplate reader (SYNERGYH4, BioTek).

### 4.11. Effect of RvD1 on the Adhesion of Microbial Cells to HaCat 

To study the effect of RvD1 on the adhesion ability of the two strains to HaCaT cells, once cells were confluent, yeast/bacterial cells (~6 Log CFU/well) were added to the wells with or without RvD1 at a concentration of 2.5 μg mL ^−1^. After 2 h of incubation at 37 °C, wells were washed with PBS, and microbial-adhered cells were recovered by accurately scraping the wells. Serial dilutions were made in PBS, and plated onto TSA or RB agar plates to count CFUs of *S. aureus* and *C. parapsilosis*, respectively, after incubation at 37 °C for 24/48 h.

### 4.12. Statistical Analysis 

The results were obtained from two or three different experiments and showed as mean ± standard deviation (SD). A one-way analysis of variance (ANOVA) followed by Dunnett’s multiple comparation test was used to compare treated to control groups. Data with *p*-values < 0.05 were considered statistically significant. 

REST software (Relative Expression Software Tool, Weihenstephan, Germany, version 1.9.12) was used to evaluate the expression gene. Fold changes greater than 2 (Log 2 (fold change) > 1) or lower than 0.5 (Log 2 (fold change) < −1) and for which the one-sample *t*-test restituted a *p* value < 0.05 were considered significantly different from 1. 

All graphics were created using the GraphPad Prism software (8.02 for Windows, GraphPad Software, La Jolla, CA, USA, www.graphpad.com accessed on 7 July 2023).

## Figures and Tables

**Table 1:** MIC, MBC, or MFC values of RvD1 against *S. aureus* and *C. parapsilosis*. In the third column, the ratios MBC or MFC/MIC are reported.

	MIC	MBC or MFC	MBC or MFC/MIC
	(μg mL^−1^)		Ratio
*S. aureus* ATCC 6538	5.0 ± 0.5	15.0 ± 0.3	3.0 ± 0.5
*C. parapsilosis* DSM 5784	10.0 ± 0.6	25.0 ± 0.02	2.5.0 ± 0.1

## Data Availability

Not applicable.

## Supplementary Materials

The following supporting information can be downloaded at: https://www.mdpi.com/article/10.3390/ijms241612876/s1. References [52,53,54,55] are cited in the supplementary materials.
