# Supplementary material for: A Re-Purposing Strategy: Sub-Lethal Concentrations of an Eicosanoid Derived from the Omega-3-Polyunsaturated Fatty Acid Resolvin D1 Affect Dual Species Biofilms
Source: Int J Mol Sci. 2023 Aug 17;24(16):12876. doi: 10.3390/ijms241612876 (PMC10454369; doi:10.3390/ijms241612876)
Supplement: Supplementary file 1 [file ijms-24-12876-s001.zip › ijms-2548303-supplementary.pdf]

**Table S1.** Gene-specific primers used for real-time RT-PCR.

| Gene name | Acronym | Primer name | Sequence (5'→3') | Melting Temperature (°C) | Reference |
|---|---|---|---|---|---|
| *16S rRNA* | *16S rRNa* | *S. aureus_16SrRNA*_F<br>*S. aureus_16SrRNA*_R | TATGGAGGAACACCAGTGGCGAAG<br>TCATCGTTTACGGCGTGGACTACC | 58 | [1] |
| *Biofilm associated protein* | *bap* | *S. aureus_bap*_F<br>*S. aureus_bap*_R | CCCTATATCGAAGGTGTAGAATTG<br>GCTGTTGAAGTTAATACTGTACCTGC | 60 | [2] |
| *Intercellular adhesion gene* | *icaA* | *S. aureus_bap*_R<br>*S. aureus_icaA*_R | GCTGTTGAAGTTAATACTGTACCTGC<br>TCAGGCACTAACATCCAGCA | 55.5 | [3] |
| *Intercellular adhesion gene* | *icaD* | *S. aureus_icaD*_F<br>*S. aureus_icaD*_R | ATGGTCAAGCCCAGACAGAG<br>CGTGTTTTCAACATTTAATGCAA | 55.5 | [3] |
| *Actin* | *Actin* | *C. parapsilosis_actin*_F<br>*C. parapsilosis_actin*_R | ACGGTATTGTTTCCAACTGGGACG<br>TGGAGCTTCGGTCAACAAAACTGG | 65 | [4] |
| *Agglutinin like-sequence 3* | *ALS3* | *C. parapsilosis_ALS3*_F<br>*C. parapsilosis_ALS3*_R | AAGTCGAGACCCACCCATTG<br>TTGTGTCCCTTTGCACTGCC | 60 | [4] |
| *Enhanced filamentous growth* | *EFG1* | *C. parapsilosis_EFG1*_F<br>*C. parapsilosis_EFG1*_R | AAGTCGAGACCCACCCAT<br>TTGTGTCCCTTTGCACTGCC | 60 | [5] |
| *Ergosterol biosynthesis enzyme* | *ERG11* | *C. parapsilosis_ERG11*_F<br>*C. parapsilosis_ERG11*_R | TGTTGCATTTGGCTGAGAAG<br>TCTGAGGGTTTCCTTGATGG | 58 | [4] |


1. Gambino, E.; Maione, A.; Guida, M.; Albarano, L.; Carraturo, F.; Galdiero, E.; Di Onofrio, V. Evaluation of the Pathogenic-Mixed Biofilm Formation of Pseudomonas aeruginosa/Staphylococcus aureus and Treatment with Limonene on Three Different Materials by a Dynamic Model. *International Journal of Environmental Research and Public Health* **2022**, *19*, 3741.
2. Cucarella, C.; Solano, C.; Valle, J.; Amorena, B.; Lasa, I.; Penadés, J.R. Bap, a Staphylococcus aureus surface protein involved in biofilm formation. *J Bacteriol* **2001**, *183*, 2888-2896, doi:10.1128/jb.183.9.2888-2896.2001.
3. Arciola, C.R.; Baldassarri, L.; Montanaro, L. Presence of icaA and icaD genes and slime production in a collection of staphylococcal strains from catheter-associated infections. *J Clin Microbiol* **2001**, *39*, 2151-2156, doi:10.1128/jcm.39.6.2151-2156.2001.
4. Maione, A.; Bellavita, R.; de Alteriis, E.; Galdiero, S.; Albarano, L.; La Pietra, A.; Guida, M.; Parrilli, E.; D'Angelo, C.; Galdiero, E.; et al. WMR Peptide as Antifungal and Antibiofilm against Albicans and Non-Albicans Candida Species: Shreds of Evidence on the Mechanism of Action. *International Journal of Molecular Sciences* **2022**, *23*, 2151.
5. Poon, Y.; Hui, M. Inhibitory effect of lactobacilli supernatants on biofilm and filamentation of Candida albicans, Candida tropicalis, and Candida parapsilosis. *Front Microbiol* **2023**, *14*, 1105949, doi:10.3389/fmicb.2023.1105949.
